# Author’s Reply

**Published:** 2010-04

**Authors:** M. S. Najar, C. L. Saldanha, K. A. Banday

**Affiliations:** Department of Nephrology, Sher-i-Kashmir of Institute of Medical Sciences, Soura, India; 1 Department of Gynecology and Obstetrics, Sher-i-Kashmir of Institute of Medical Sciences, Soura, India; 2 SKIMS Medical College, Bemina, Srinagar, J and K, India

Sir,

We agree with the readers of our article “Approach to Urinary Tract Infection” when they point out that it is important to treat both gonorrhoea and Chlamydia in a patient of urethritis simultaneously. Gonorrhoea need not be treated if excluded after proper testing and investigation. Importance of *Chlamydia trachomatis* infection also lies in the fact that it is the most common reportable infectious disease in the United States of America.[1] Azithromycin is now recommended as a primary rather than alternative treatment in pregnant women with *Chlamydia trachomatis* infection. This change occurred because of recent evidence supporting azithromicin as safe and effective during pregnancy.[2]

Both Gonococal and Chlamydial infections are sexually transmitted diseases (STDs) and the standard practice is to recommend treatment of sex partners of the patients of these infections to decrease the risk of reinfection. The primary goal is for the patient’s sex partner to be examined by a physician for testing, treatment and education. However, there may be clinical situations in which this cannot be accomplished (e.g., because of patient, partner or resource limitations). In these circumstances, the Center for Disease Control recommends that physicians consider using “expedited partner treatment.” This is the practice of treating sex partners of persons diagnosed with an STD without medical evaluation or prevention counseling by providing the patient with appropriate treatment to administer to his or her partner.[3]
